# Landscape features influence gene flow as measured by cost-distance and genetic analyses: a case study for giant pandas in the Daxiangling and Xiaoxiangling Mountains

**DOI:** 10.1186/1471-2156-11-72

**Published:** 2010-07-23

**Authors:** Lifeng Zhu, Xiangjiang Zhan, Tao Meng, Shanning Zhang, Fuwen Wei

**Affiliations:** 1 Key Laboratory of Animal Ecology and Conservation Biology, Institute of Zoology, Chinese Academy of Sciences, Datunlu, Chaoyang District, Beijing, 100101, People's Republic of China; 2 China Wildlife Conservation Association, No 18, Hepingli East Street, Beijing, 100714, People's Republic of China

## Abstract

**Background:**

Gene flow maintains genetic diversity within a species and is influenced by individual behavior and the geographical features of the species' habitat. Here, we have characterized the geographical distribution of genetic patterns in giant pandas (*Ailuropoda melanoleuca*) living in four isolated patches of the Xiaoxiangling and Daxiangling Mountains. Three geographic distance definitions were used with the "isolation by distance theory": Euclidean distance (EUD), least-cost path distance (LCD) defined by food resources, and LCD defined by habitat suitability.

**Results:**

A total of 136 genotypes were obtained from 192 fecal samples and one blood sample, corresponding to 53 unique genotypes. Geographical maps plotted at high resolution using smaller neighborhood radius definitions produced large cost distances, because smaller radii include a finer level of detail in considering each pixel. Mantel tests showed that most correlation indices, particularly bamboo resources defined for different sizes of raster cell, were slightly larger than the correlations calculated for the Euclidean distance, with the exception of Patch C. We found that natural barriers might have decreased gene flow between the Xiaoxiangling and Daxiangling regions.

**Conclusions:**

Landscape features were found to partially influence gene flow in the giant panda population. This result is closely linked to the biological character and behavior of giant pandas because, as bamboo feeders, individuals spend most of their lives eating bamboo or moving within the bamboo forest. Landscape-based genetic analysis suggests that gene flow will be enhanced if the connectivity between currently fragmented bamboo forests is increased.

## Background

Gene flow, in the form of effective individual gene movement within and between populations, is one of the most important factors for maintaining genetic diversity within a species and counteracting the negative effects of habitat fragmentation [1,2]. Landscape connectivity [3,4], based on landscape features, is critical for the persistence of spatially structured populations. Recently, studies have shown that gene flow depends heavily on individual behavior within certain landscapes (e.g. in populations of terrestrial Mediterranean snakes [5], roe deer (*Capreolus capreolus*) [6], or mountain vizcacha (*Lagidium viscacia*) [7]). Therefore, landscape genetics, a subdiscipline of population genetics, has been introduced to quantify geographic distributions of genetic patterns (e.g. clines [8]), isolation by distance, and correlations between genetic patterns and landscape variables [9].

The most common methodology adopted for landscape genetics studies has been the comparison of geographic and genetic distance matrices to describe the geographical structure of genetic variability, at a fine spatial scale, within a population. The Euclidean distance (EUD) was the first metric used for these correlation matrices, and is currently the most frequently used metric for quantifying geographic distance between individuals [10-13]. The EUD distance has proven to be effective in describing individual movement in relatively homogeneous or small-scaled habitats. However, most animals live in heterogeneous habitats, and individual movement is greatly influenced by landscape elements [14,15] that introduce bias into results based on a EUD measure. The least-cost path distance (LCD), which defines a measure of landscape connectivity, was, therefore, introduced as a more suitable means for assessing the inferred effects of landscape structure on gene flow [16-19]. The least-cost path avoids landscape regions that are more resistant to movement and prefers paths through permeable features. LCD can be approximated by the path that minimizes the sum of the 'costs' of every raster cell traversed along the path [20,21]. Costs are defined by the geographical information embedded in the landscape and the behavioral and ecological characteristics of the species being evaluated [22]. Models of functional connectivity, created using cost distance analysis, can be tested by analyzing highly variable genetic markers to determine potential movement and dispersal throughout a landscape [16,19,23,24]. Estimates of cost distances are based on major features that influence individual movement or dispersal (such as the distribution of wooded habitat for roe deer [16], basking habitat for timber rattlesnake hibernacula [19]) or a landscape resistance model [25]. Factors such as topographic (altitude, gradient, and slope) or anthropogenic factors (road construction, human residence) may also influence the movement or dispersal of individuals. Animal movement is modeled as a trade-off that mitigates many factors [19,26] and reflects the process of habitat selection.

**Figure 1:**
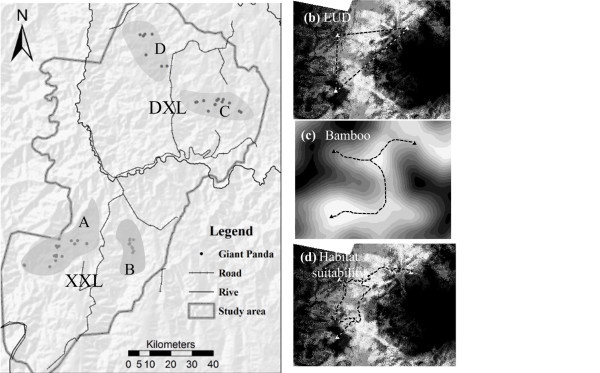
**The study areas and three definitions of geographical distance**. Figure 1 (a): A includes Mianning-Yele, Jiulong-Wanba, and Shimian-Caoke. B includes Shimian-Liziping. C includes Yingjing-Shizi and Wawu mountains. D includes Yingjing-Xinmiao, Sanhe, and Jianzheng. The right corner shows the extant distribution of giant pandas across the entire country. Figure 1 (b): The Euclidean distance (EUD) between any two individuals. This is the shortest straight line separating two individuals. Figure 1 (c), (d): The least cost distance (LCD) between any two individuals, where cost is calculated as the sum of the values (bamboo or habitat suitability, respectively) of each pixel along the paths connecting individuals. Dark indicates the cost extent of the panda across the habitat.

The giant panda (*Ailuropoda melanoleuca*) is often cited as one of the most endangered mammals in the world [27]. Currently, the species is confined to six fragmented mountain habitats at the edge of the Tibetan Plateau [27]. Among the fragmented habitats, the Xiaoxiangling (XXL) and Daxiangling (DXL) forests are the smallest, with a combined population of around 60 individuals [28]. These are also the most fragmented habitats because the Dadu River and National Road 108 have divided the habitat into four major patches (Figure 1), and a strong human presence from the local residents along the river and the road disturbs the habitat. Giant pandas are very large and elusive mammals. Each individual has a home range spanning an area of 3-7 km^2^, where it spends most of its time. Females tend to forage only within their home range [27], whereas the home ranges of males may overlap. The dispersal distance can be several kilometers or more [27,29]. However, individual movement is complicated, determined by many landscape and environmental factors, such as altitude, slope, and human disturbance [30,31]. Pandas prefer well-wooded slopes with an almost continuous forest canopy [29]. However, until recently, there have been no quantitative studies describing the relationship between landscape features and the behavior of the giant panda, particularly in terms of gene flow within the population. We selected the two methods to measure LCD paths in this study. One LCD path was defined by the bamboo resources, the main food of the giant panda. A second LCD path was defined by habitat suitability, a complicated landscape ecological model that integrates several environmental variables.

The classic EUD distance and two LCD distance measures were applied in a landscape genetic analysis of the giant panda population in the DXL and XXL mountains. These models tested the hypothesis that landscape features influence gene flow in the giant panda population.

## Results

### Genetic diversity and population structure

A total of 136 genotypes were obtained from 192 fecal samples and one blood sample, yielding 53 unique individual genotypes. The consistency between genotypes was checked according to standard replication criteria. Using the formulae [32], the mean genotype error rate per locus was estimated to be 0.16%, and the genotype identification error rate across nine loci was estimated to be 1.4%. Therefore, we expected at most two incorrect genotype identifications among these produced, which have the potential to upwardly bias our population estimate slightly. The combination of the nine chosen loci can only characterize the genotypes of full siblings as identical, by chance, with a probability of 0.00074. In total, 53 individuals were identified, including 11 females and 9 males in Patch A, 6 females and 6 males in Patch B, 7 females and 7 males in Patch C, and 4 females and 3 males in Patch D. The average allele number *A *was between two and seven alleles per locus across all samples (Table 1). The average *He *fell in the range 0.56-0.63, but *Ho *values were slightly higher (0.66-0.71), generating slightly negative *Fis *values (average *Fis *= -0.013, non-significant). The *Ho *values were higher than the *He *values, in agreement with results from a previous study of the same region [33]. No evidence was found for null alleles, stuttering, or allele dropout in each locus by the program Microchecker, at a confidence level of 100%.

**Table 1:** Summary of basic population genetic analysis for the four populations

Region	Patch (Populations)	Patch Size(km^2^)	Euclidian Distance(km)	Locus	No. of Alleles	*Ho*	*He*
Xiaoxiangling (XXL)	A (n = 20)	450		Ame-*μ*5	6	0.900	0.795
			Minimum: 0.1	Ame-*μ*10	7	0.944	0.810
				Ame-*μ*26	2	0.650	0.512
			Mean: 8.2	Ame-*μ*15	4	0.800	0.636
				Ame-*μ*16	5	0.790	0.623
			Maximum: 31.1	Ame-*μ*13	4	0.739	0.755
				Ame-*μ*22	3	0.400	0.344
				AY161179*	6	0.550	0.803
				AY161195	4	0.600	0.545
				All loci	4.6	0.708	0.630

	B (n = 12)	230		Ame-*μ*5	6	0.833	0.837
			Minimum: 0.1	Ame-*μ*10	4	0.750	0.757
				Ame-*μ*26	2	0.583	0.518
			Mean: 2.9	Ame-*μ*15	3	0.750	0.565
				Ame-*μ*16	3	0.500	0.416
			Maximum: 6.5	Ame-*μ*13	4	0.818	0.727
				Ame-*μ*22	3	0.500	0.420
				AY161179	4	0.917	0.725
				AY161195	3	0.636	0.671
				All loci	3.7	0.699	0.599

Daxiangling (DXL)	C (n = 14)	320		Ame-*μ*5	5	0.857	0.720
			Minimum: 0.2	Ame-*μ*10	6	0.786	0.807
				Ame-*μ*26	3	0.714	0.627
			Mean: 8.0	Ame-*μ*15	4	0.571	0.558
				Ame-*μ*16	4	0.692	0.649
			Maximum: 21.8	Ame-*μ*13	4	0.786	0.664
				Ame-*μ*22	3	0.571	0.500
				AY161179	5	0.500	0.603
				AY161195	4	0.429	0.545
				All loci	4.2	0.656	0.608

	D (n = 7)	350		Ame-*μ*5	2	0.714	0.495
			Minimum: 1.1	Ame-*μ*10	5	0.85714	0.758
				Ame-*μ*26*	3	0.857	0.659
			Mean: 10.4	Ame-*μ*15	3	0.286	0.473
				Ame-*μ*16	4	0.500	0.788
			Maximum: 19.4	Ame-*μ*13	4	1.000	0.780
				Ame-*μ*22	2	0.143	0.143
				AY161179	3	0.857	0.692
				AY161195	3	0.833	0.667
				All loci	3.2	0.672	0.561

*H*_O_, observed heterozygosity; *H*_E_, heterozygosity expected under Hardy-Weinberg equilibrium. The asterisks (*) indicate significant (*P *< 0.05) departures from Hardy-Weinberg equilibrium.

In XXL, there were two individuals living in Luding county. The mean distance to Patch A or B was greater than 70 km. Therefore, because the sample size was small and the mantel test biased, we excluded these individuals in the following analysis.

### Correlations between genetic and geographical distances as a function of raster size

**Figure 2:**
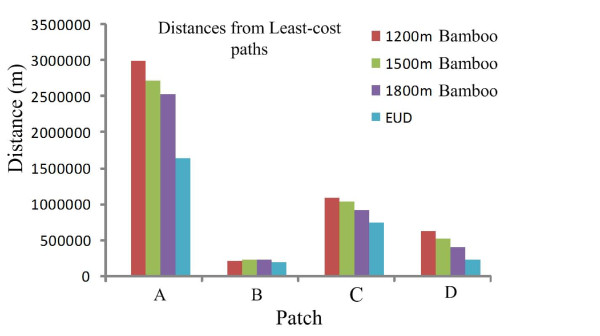
**Least-cost pathways for giant pandas in four patches**: mapped using a neighborhood radius of 1500 m and a raster cell size of 90 m.

The numerical model was affected by the choice of radius (from 1200 to 1800 m). Small radii tended to yield larger cost distances (for example, Figure 2) because smaller radii yield a finer level of informational detail at each pixel. The variation in cost distance was small in Patch B (Figure 2), which may be due to the small patch area and the concentrated distribution of individuals. At radii of 1200 and 1500 m, positive correlation indices between genetic and LCD distances tended to be larger than the correlation indices calculated using the EUD, and were more negative in Patch C. However, no consensus trend was observed for analysis performed with a radius of 1800 m in Patches B and D. Thus, for the biological mean (home range), a conservative radius of 1500 m was chosen for subsequent analysis.

**Figure 3:**
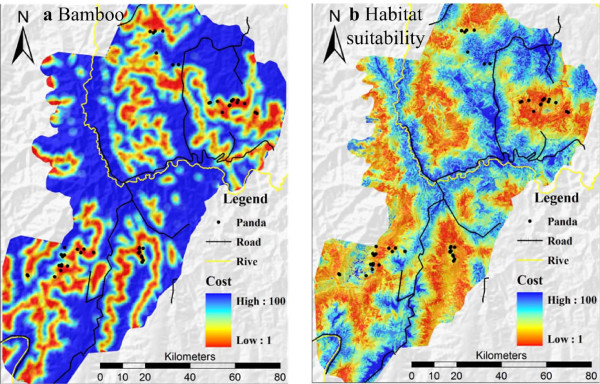
**The classification of satellite images and the cost value grids for two LCD models in the study region**. The black dot represents the giant panda sightings.

The correlation indices between genetic and pairwise least-cost paths were different for different raster cell sizes because LCD paths, computed using friction maps of different resolutions, appeared to differ in shape and length. The permeability of the local landscape structures depended on the resolution of the friction maps used, which has been observed in studies of the American marten (*Martes americana*) [24]. Generally, analysis using grid cell sizes larger than 500 m yielded a decrease in the correlation indices (data not shown). Thus, we compared the effects of cell size variation for sizes smaller than 250 m. The standard deviation (SD) of the correlation indices calculated by habitat suitability analysis was larger than those based on bamboo resources calculated with a radius of 1500 m (Table 2), indicating that this type of LCD path may have a larger influence on raster cell size. Correlations based on a raster cell size of 90 m (Figure 3) are displayed in Figures 3 and 2 to illustrate the relatively large correlation index (except for Patch D). Mantel tests showed that most correlation indices calculated using two LCD, particularly for bamboo resources calculated using different raster cell sizes, were slightly larger than those calculated using EUD, with the exception of Patch C (which gave a more negative correlation index than the correlation index calculated using EUD). In Patch D, the variation in correlation index calculated using the LCD of habitat suitability was large. In addition, a significant relationship was only found in Patch A. Patch C showed a negative but insignificant relationship (*P *> 0.05) for a radius of 1500 m.

**Table 2:** Correlation between genetic and geographic distances (Mantel test).

Patch	Distance	Raster cell size	Correlation index of Mantel test (*P *value given in brackets)			
**A**	EUD		*0.261 (0.008)*			
			
			Bam (1200 m)	Bam (1500 m)	Bam (1800 m)	Habitat
			
	LCD	30 m	*0.288 (0.001)*	*0.267 (0.001)*	*0.260 (0.002)*	*0.268 (0.010)*
		60 m	*0.286 (0.001)*	*0.271 (0.001)*	*0.261 (0.002)*	*0.275 (0.009)*
		90 m	*0.287 (0.001)*	*0.265 (0.002)*	*0.268 (0.001)*	*0.275 (0.008)*
		120 m	*0.278 (0.001)*	*0.267 (0.001)*	*0.259 (0.002)*	*0.264 (0.010)*
		250 m	*0.279 (0.001)*	*0.263 (0.002)*	*0.278 (0.000)*	*0.278 (0.007)*
		**SD**	0.0047	0.0030	0.0080	0.0058

**B**	EUD		0.067 (0.286)			
	LCD	30 m	0.075 (0.282)	0.076 (0.274)	0.047 (0.357)	0.080 (0.247)
		60 m	0.081 (0.264)	0.067 (0.292)	0.043 (0.035)	0.070 (0.287)
		90 m	0.089 (0.249)	0.102 (0.201)	0.057 (0.326)	0.118 (0.168)
		120 m	0.064 (0.313)	0.078 (0.264)	0.035 (0.384)	0.056 (0.323)
		250 m	0.074 (0.286)	0.090 (0.241)	0.048 (0.347)	0.075 (0.258)
		**SD**	0.0092	0.0136	0.0080	0.0232

**C**	EUD		-0.153 (0.112)			
	LCD	30 m	*-0.211 (0.046)*	-0.186 (0.063)	-0.138 (0.131)	-0.168 (0.092)
		60 m	-0.197 (0.062)	-0.184 (0.062)	-0.139 (0.126)	-0.173 (0.085)
		90 m	*-0.211 (0.047)*	-0.192 (0.054)	-0.139 (0.121)	-0.172 (0.078)
		120 m	*-0.215 (0.041)*	-0.193 (0.057)	-0.142 (0.119)	-0.147 (0.109)
		250 m	-0.203 (0.052)	-0.139 (0.127)	-0.133 (0.142)	-0.151 (0.105)
		**SD**	0.0073	0.0226	0.0033	0.0123

**D**	EUD		0.212 (0.147)			
	LCD	30 m	0.262 (0.167)	0.268 (0.122)	0.227 (0.139)	0.251 (0.122)
		60 m	0.284 (0.112)	0.267 (0.131)	0.196 (0.166)	0.180 (0.172)
		90 m	0.281 (0.111)	0.283 (0.112)	0.226 (0.141)	0.195 (0.163)
		120 m	0.280 (0.113)	0.283 (0.115)	0.227 (0.138)	0.211 (0.148)
		250 m	0.237 (0.129)	0.264 (0.121)	0.229 (0.130)	0.238 (0.126)
		**SD**	0.0198	0.0092	0.0140	0.0294

'Bam (1200)' indicates that the least-cost path defined by bamboo resources with a neighborhood radius of 1200 m. Bam (1500) and Bam (1800) are similarly defined. 'Habitat' indicates that the least-cost paths are based on habitat suitability analysis. '**SD**' indicates the standard deviation. The numbers in italics indicate significant mantel tests (*P *< 0.05).

### The effect of the low sample number in IBD analyses

The range of power for our test is 0.08-0.34 (Table 3). The value of the power in the DXL level is still low, because of the low relationship index. Assuming animals were randomly distributed, the required sample size to reach adequate power for a low index was very large and unrealistic for these researches of wild rare animals. In addition, the average power of statistics based on 100 simulation data (7 individuals) was 0.11 (95% confidence region: 0.051-0.020) and therefore the power of the test for Patch D (the smallest sample size) fell within this confidence region (Table 3).

**Table 3:** Statistical power of our tests and correlations between genetic and EUD distances (Mantel test) between patches.

Region	Sample	Correlation index (P value)	Power of Test*
Patch			

A	18	*0.261 (0.008)*	0.25

B	12	0.067 (0.286)	0.08

C	14	-0.153 (0.112)	0.14

D	7	0.212 (0.147)	0.13

Mountain			

XXL	30	0.217(*0.000*)	0.34

DXL	21	0.078(0.193)	0.10

• Power of test: 1-*β *(type II error); *α *= 0.05 level.

### The spatial genetic structure (decreasing the gene flow)

**Figure 4:**
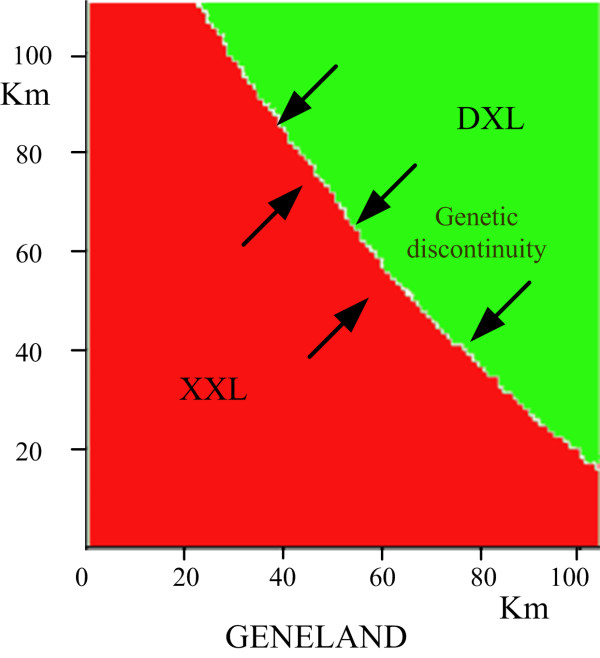
**The population structure in the Xiaoxiangling and Daxiangling Mountains**.

The populations of XXL and DXL were consistently of two different genetic clusters (K = 2, Figure 4), and a clear genetic discontinuity was found between them. In addition, we re-conducted the Mantel test at a mountain scale (XXL and DXL), however, we only found the significant IBD pattern in the XXL region (Table 3).

## Discussion

### Landscape features partially influence gene flow within the patch level

Giant pandas are large and elusive mammals, and spend most of their life in a home range. Currently, there are approximately 1600 pandas in the wild, restricted to 24 fragmented forests [28]. The individual size of each population is small, especially in our study area.. However, this gives us a chance to sample more extensively and to investigate fine-scale landscape genetics. In fact, we have sampled most of the local populations [34]. Moreover, we have to acknowledge the effect of our low sample number on the mantel test as the statistical power for our analysis was low. Both sample size and the random distribution of giant pandas may have influenced the power of our test and some caution in our interpretation is warranted. This phenomenon might be a common one in fine-scale analyses involving large or elusive endangered mammals in habiting fragmented habitats [35].

On the whole, our results partially support the hypothesis that geographical landscape features influence gene flow of the giant panda (Table 2). These results might be closely linked to the biological characteristics and behavior of pandas. (1) The giant panda, a bamboo feeder, spends most of his/her life eating bamboo or moving within a bamboo forest, and the food intake of an adult panda is enormous, 10-18 kg (av. 12.5) per day [29]. (2) A previous radio tracking study showed that pandas prefer to move within well-wooded forests with a continuous forest canopy [27,31], because bamboo grows more densely in open spaces than it does beneath a canopy, and the stalks are drier and more stunted, which affects the nutritional content of the bamboo [29]. In continuous bamboo forests, pandas can reduce energy expenditures related to seeking food [29]. (3) Lastly, movement and dispersal of giant pandas is complicated, and is influenced by many environmental variables [30,31]. In our habitat suitability analysis, we have integrated 11 relevant environmental variables (Table 4).

**Table 4:** The environmental variable used in the model of ENFA

Environmental variable	Description of the variable
ELEV	Elevation of the study area
ELEV-SD	Standard deviation of altitude in a 800-m radius
SLOP	Slop of the study area
SLOP-SD	Standard deviation of the slop in a 800-m radius
EASTNESS	Average eastness in a 800-m radius (Sine of the aspect)
NORTHNESS	Average northness in a 800-m radius (Cosine of the aspect)
DIST-RES	Distance to the resident
DIST-ROA	Distance to the main road
FORE-FQ	Forest frequency in the 800-m radius
SHRB-FQ	Shrub frequency in the 800-m radius
DIST-LAN	Distance to the land(non-forest)

Our study demonstrated that the correlations between landscape features and gene flow (dispersal) were enhanced by the presence of habitat types preferred by the species, in agreement with other studies. For example, the mobility of forest species is favored by wooded landscapes, and correlation coefficients between dispersal data and the LCD measures were slightly stronger than with Euclidean distance measures (see, for example, roe deer, [16]; martens, [17]; mountain vizcacha, [7]). A previous study [19] found a significant positive correlation between genetic differentiation and a cost-based distance metric adjusted to include the quantity of potential basking habitat between hibernacula.

### Geographic measurements that best explain the relationship between landscape features and gene flow

A comparison of several geographic measurements showed that the bamboo resources measure yielded slightly more influence on gene flow, as indicated by the significantly larger positive correlation indices calculated for this measure compared to the EUD measure in Patches A and D (non-parameter test, *P *< 0.05). The effects of habitat suitability depended more strongly on raster cell size and yielded larger SDs (standard deviation) between correlation indices than the effects of bamboo resources. No significant changes were observed in the Mantel test after using LCD (from non-significant to significant, Table 2). The effects of landscape features on gene flow were different in the different patches.

These patterns may be rationalized by several observations. First, EUD is defined by the simplest straight line between individuals, presenting an idealized travel route that is unrealistic for giant pandas in most circumstances. The movement of giant pandas is complicated by many landscape and environment factors [30,31]. Food (bamboo resources) may have the largest influence on behavior [27,29]. The model for habitat suitability integrated 11 variables, but was biased to favor more realistic movement paths for giant pandas. Therefore, a simple model based on an LCD defined by bamboo resources performed slightly better than a model based on an EUD measure. Second, if the connectivity between bamboo resources was high, the cost distances defined by bamboo resource distributions did not vary significantly with raster cell size. The inter-habitat suitability map, on the other hand, was more fragmented. Raster cell size produced large variations in the cost-distance path, such as those seen in Patch D. Third, considering the relatively small size of each patch, the mantel tests for IBD patterns based on EUD paths and two types of LCD paths, with a radius of 1500 m, gave similar results (Table 2). For example, the area of Patch B is small, and giant pandas live mainly in one large gully. The paths of EUD and two LCD are similar. Last, giant pandas may move over long distances, and genes may flow within overlapping home ranges [27], which can bias the IBD gene flow pattern within the connected bamboo resources.

### Habitat fragmentation and gene flow of giant pandas between patches

Our results show that habitat loss and fragmentation might have decreased gene flow of giant pandas. Major river courses (the Dadu River) might have played an important role in shaping boundaries of groups in giant panda, notably the significant two genetic clusters, XXL and DXL (Figure 4). Moreover, human activity along the river and the presence of roads has further lead to habitat loss and fragmentation across the XXL and DXL mountains (Figure 1). However, the genetic boundaries and spatial dynamics in these regions need further investigation.

We found a significant IBD pattern in the XXL region, especially in Patch A (Table 2 and 3). According to our wild investigation and the third national survey of giant pandas [28], the mean altitude of giant panda activity in Patch A is the highest (3500.74 m ± 219.06), and the major bamboo is *Bashania spanostachya Yi*. The forest and food (bamboo) resource fragmentation is the most serious threat to habitat, and has limited the panda's distribution to within three major ditches, the Niuchang, the Dayang, and the Caimagu ditches. Thus, limited food resources may lead to a non-random distribution of individuals. In addition, there was no significant pattern found in the DXL region (Table 3). In the DXL region individuals live at a low range of altitudes and have sufficient resources (*Yushania lineolata Yi and Bashania spanostachya Yi*). Thus, they are able to move freely about the landscape, reflecting the partial random distribution.

## Conclusions

Here we found that landscape features might influence gene flow of giant pandas across two scales. First, within patches, the use of an LCD measure improves the model for individual movement and gene flow by broadening the geographic measurements that are integrated into one or more important ecogeographical variables, such as food resources (bamboo). However, some uncertainty was introduced by the size of the neighborhood radius defined in the numerical model and by the raster cell size. Although use of a complicated model or several parameters to describe landscape ecology increases uncertainty in the current model implementations, improvements can be made by integrating additional ecological factors, including intra-specific interactions and kin and resource competition. Giant panda research has yet characterized larger populations, which would decrease the bias inherent in small population sizes. If using LCD methods, the effects of neighborhood radius and raster cell size on least-cost path approaches should be rigorously investigated. Second, at a broader inter-patch scale, natural barriers and human activity along the river might have further decreased gene flow and led to habitat fragmentation and subsequent population differentiation of giant pandas in these regions. Therefore, the landscape genetic analysis presented here suggests that it is vital to connect currently fragmented habitats and increase the connectivity of bamboo resources within a habitat to restore population viability of the giant panda in these regions. For these small isolated populations reintroductions will be an effective strategy.

## Methods

### Sample collection

In total, 192 fecal samples and one blood sample were collected in each of the four patches of the DXL and XXL mountains (Figure 1a) between March and October, 2005. The mean distance between patches was 76 km. Field staff performed a 'zig-zag' search for panda feces, gully by gully and slope by slope, in an altitude range of 2,000 and 3,900 m. Most samples were less than two weeks old, as judged by the status of the mucosal outer layer of the feces. All samples were GPS positioned. Up to five grams of feces were peeled from the outer layer and stored in 99% ethanol.

### DNA extraction and amplification

DNA was extracted from feces with standard controls [36]. Eighteen giant panda microsatellite genetic loci [37] and three redesigned loci [38] were initially assessed, and nine loci (*Ame*-05, *Ame*-10, *Ame*-13, *Ame*-15, *Ame*-16, *Ame*-26, *Ame*-22, AFAY161179, and AY161195) were selected for this study on the basis of PCR efficiency, polymorphisms, and yield. To obtain reliable genotypes, a modified multi-tube approach [39] was used as follows: Fifty cycles of PCR amplification were carried out simultaneously for up to four loci, with combinations selected based on fragment size, *T*_m_, and fluorescent dye (FAM, TET, or HEX), using the QIAGEN Mutliplex PCR kit according to the manufacturer's protocol at optimized annealing temperatures. Products were resolved using an ABI 377 prism automated sequencer, and analyzed using GeneScan v3.1.2 and Genotyper 2.5 (Applied Biosystems). Sex identification was carried out according to previously described methods [40]. A species-specific sexing primer pair ZX1 was designed to amplify a 210 bp region of the Y chromosome of the giant panda. PCR and cycling conditions were similar to those used for microsatellite amplification. Each sample was amplified three times with ZX1, and products were separated by electrophoresis on a 2.0% agarose gel. A sample was identified as male if at least two experiments showed the 210 bp SRY band, and as female if no bands were produced.

Genotyping errors are frequently encountered in noninvasive genetic analysis using fecal samples [39,41], and pre-selection of samples and rigorous laboratory procedures must be followed to produce accurate genotypes. As part of this process, we conducted mitochondrial DNA analysis for species verification, and our microsatellite genotyping protocol followed the criteria [39]. Genotype error rates were estimated using a mathematical approach [32]. The software GIMLET was used to calculated the probabilities of identity (P_(ID) _and P_(ID-sibs)_) to quantify the efficacy in discriminating the nine loci in combination.

### Genetic diversity and pairwise individual genetic distances

Genetic diversity was measured as the mean number of alleles per locus (*A*), observed heterozygosity (*H_O_*), and expected heterozygosity (*H_E_*) [42]. Wright's *F *statistics were estimated [43]. We also calculated the deviations from the Hardy-Weinberg equilibrium for each locus of each population. Analysis was performed using Arelquin v3 (Excoffier and Schneider 2005). The presence of null alleles, stuttering, and small allele dominance was tested using Microchecker [44]. Genetic distances between individuals, *a_r_*, were defined [11] and computed using SPAGeDI [45].

### Landscape features and four geographical distances

#### Euclidean distance

The Euclidean distance, as the traditional predictor of genetic difference between populations, was calculated using geographic straight-line distances between each pair of individuals using the ArcGIS 9.0 software (Figure 1b).

#### Least cost distance based on food resources (bamboo)

The map of the bamboo distribution was imported from a previous study [28] and our field survey. A grid map of the bamboo distribution was made using five raster cell sizes (30, 60, 90, 120, and 250 m), and cells were assigned as either containing or excluding bamboo. The density of bamboo was averaged by a 1500 m radius circular moving window. This neighborhood radius was chosen based on the giant panda home range size (3-7 km^2^) [27,29]. Different radii were tested (1200 and 1800 m) to gauge the effect of neighborhood size on LCD analysis. A raster map of bamboo density assigned cost values to each cell in the range 0 to 100. In this map, cells with a bamboo density of 0 were assigned a cost value of 100, indicating the maximum travel cost of a panda through that region, and a cost value of 1 was assigned to cells with a bamboo density of 100, which was the minimum travel cost. In this way, a resistance or travel cost grid map of panda movement was calculated. The travel cost map permitted calculation of the least-cost distance between pairs of panda individuals using PATHMATRIX [46] in Arcview3.2 (Figure 1c and 2a).

#### Least cost distance based on habitat suitability

The Ecological Niche Factor Analysis (ENFA, [47]) model identifies a set of uncorrelated factors that accounts for the information by comparing the distributions of environmental variables and the population distribution dataset across the surveyed geographical area. One factor, Marginality, was defined as the ecological distance between the species optimum and the mean habitat within a reference area. A second factor, Specialization, was defined as the ratio of the ecological variance in mean habitat to the variance observed for the focal species. With these factors, a habitat suitability map was plotted using the medians algorithm. Habitat suitability values for the giant panda were defined on the range 0 to 100. Higher values corresponded to higher habitat quality. ENFA analysis was performed using the BIOMAPPER3.1 [48] software. Habitat suitability was computed with 11 ecogeographical variables described in previous studies [27,29] (Table 4), including three categories of environmental descriptors: (1) the topographical variables ELEV, ELEV-SD, SLOP, SLOP-SD, EASTNESS, and NORTHNESS; (2) the biological variables FORE-FQ and SHRB-FQ; and (3) the anthropogenic variables DIST-RES, DIST-ROA, and DIST-LAN. These environmental variables were derived from satellite images, topography, and the road network GIS database, digitally represented in GIS (ArcGIS 9.0) as raster maps. Using the ENFA method, we calculated the habitat suitability index (HSI) for every cell in the study area and assigned 1 to those cells with an HSI of 100, and assigned 100 to those cells with an HSI of 0. This map yielded a map of travel cost based on habitat suitability. Environmental variables were derived from satellite images, topography, and the road network GIS database, digitally represented in GIS (ArcGIS 9.0) as raster maps. The HSI was computed from the ENFA. 100 minus the value of the HSI gave the value of cost for LCD analysis. The LCD was computed using PATHMATRIX [46] in Arcview3.2 (Figure 1d and 2b). LCD was calculated with different cell sizes, 30 m, 60 m, 90 m, 120 m, and 250 m.

### Relationship between genetic and geographic distances

To test the effects of landscape features on gene flow within the giant panda population, we compared the matrix of pairwise genetic distances with four matrices of geographical distances. The resulting correlations were evaluated by Mantel tests implemented in GENALEX 6.2 [49]. *P *values were obtained using a permutation procedure (10,000 permutations).

In order to evaluate the effect using a low number of individuals in our IBD analyses, we used Gpower 3.1 http://gpower.software.informer.com/3.1/ to determine the power of the test. In addition, IBDsim [50] was used to generate simulated genetic data to assess confidence for the above test. IBDsim uses a coalescent algorithm to derive various IBD models with continuous or discrete subpopulations. For nine microsatellite loci the number of alleles allowed in the model was 15 and a generalized stepwise mutation (GSM) model with a 5 × 10^-4 ^mutation rate was chosen. We conducted 100 simulations with small population size (according to the result of the individual indentified in each patch).

### Spatial genetic cluster analysis

Geneland is a computer package that allows to make use of georeferenced individual multilocus genotypes to infer the number of populations and the spatial location of genetic discontinuities between populations [51]. We ran the MCMC five times (to verify the consistency of the results), allowing K to vary, with the following parameters: 500,000 MCMC iterations, maximum rate of Poisson process fixed to 200, minimum K fixed to 1, maximum K fixed to 8. We used the Dirichlet model as a model for allelic frequencies as it has been demonstrated to perform better than any alternative model. We then inferred the number of populations in our sample from the modal K of these five runs, and ran it an additional several times with K fixed to this number.

## Abbreviations

XXL: The Xiaoxiangling Mountains; DXL: The Daxiangling Mountains; EUD: The Euclidean distance; LCD: Least-cost path distance; ENFA: Ecological Niche Factor Analysis.

## Authors' contributions

FW designed the research. LZ conducted the lab work. LZ, XZ, TM, SZ and FW analyzed the data. LZ and TM conducted the fieldwork. LZ, FW, XZ wrote the paper. All authors discussed the results and commented on the manuscript. All authors read and approved the manuscript.
